# Radiomics analysis of baseline computed tomography to predict oncological outcomes in patients treated for resectable colorectal cancer liver metastasis

**DOI:** 10.1371/journal.pone.0307815

**Published:** 2024-09-11

**Authors:** Emmanuel Montagnon, Milena Cerny, Vincent Hamilton, Thomas Derennes, André Ilinca, Mohamed El Amine Elforaici, Gilbert Jabbour, Edmond Rafie, Anni Wu, Francisco Perdigon Romero, Alexandre Cadrin-Chênevert, Samuel Kadoury, Simon Turcotte, An Tang

**Affiliations:** 1 Centre de recherche du Centre hospitalier de l’Université de Montréal (CRCHUM), Montréal, QC, Canada; 2 Department of Radiology, CISSS des Laurentides, Hôpital de Saint-Eustache, Saint-Eustache, QC, Canada; 3 Department of Radiology, Radiation Oncology and Nuclear Medicine, Université de Montréal, Montréal, QC, Canada; 4 MedICAL Laboratory, Polytechnique Montréal, Montréal, QC, Canada; 5 Division of Internal Medicine, Department of Medicine, Hôpital du Sacré-Cœur-de-Montréal, Montréal, QC, Canada; 6 Montreal AI Hub, Ericsson Canada, Montréal, QC, Canada; 7 Department of Medical Imaging, CISSS Lanaudière, Université Laval, Joliette, Québec, Canada; 8 Hepatopancreatobiliary and Liver Transplantation Division, Department of Surgery, Centre Hospitalier de l’Université de Montréal (CHUM), Montréal, QC, Canada; 9 Department of Radiology, Centre Hospitalier de l’Université de Montréal (CHUM), Montréal, QC, Canada; Queen’s University, CANADA

## Abstract

**Objective:**

The purpose of this study was to determine and compare the performance of pre-treatment clinical risk score (CRS), radiomics models based on computed (CT), and their combination for predicting time to recurrence (TTR) and disease-specific survival (DSS) in patients with colorectal cancer liver metastases.

**Methods:**

We retrospectively analyzed a prospectively maintained registry of 241 patients treated with systemic chemotherapy and surgery for colorectal cancer liver metastases. Radiomics features were extracted from baseline, pre-treatment, contrast-enhanced CT images. Multiple aggregation strategies were investigated for cases with multiple metastases. Radiomics signatures were derived using feature selection methods. Random survival forests (RSF) and neural network survival models (DeepSurv) based on radiomics features, alone or combined with CRS, were developed to predict TTR and DSS. Leveraging survival models predictions, classification models were trained to predict TTR within 18 months and DSS within 3 years. Classification performance was assessed with area under the receiver operating characteristic curve (AUC) on the test set.

**Results:**

For TTR prediction, the concordance index (95% confidence interval) was 0.57 (0.57–0.57) for CRS, 0.61 (0.60–0.61) for RSF in combination with CRS, and 0.70 (0.68–0.73) for DeepSurv in combination with CRS. For DSS prediction, the concordance index was 0.59 (0.59–0.59) for CRS, 0.57 (0.56–0.57) for RSF in combination with CRS, and 0.60 (0.58–0.61) for DeepSurv in combination with CRS. For TTR classification, the AUC was 0.33 (0.33–0.33) for CRS, 0.77 (0.75–0.78) for radiomics signature alone, and 0.58 (0.57–0.59) for DeepSurv score alone. For DSS classification, the AUC was 0.61 (0.61–0.61) for CRS, 0.57 (0.56–0.57) for radiomics signature, and 0.75 (0.74–0.76) for DeepSurv score alone.

**Conclusion:**

Radiomics-based survival models outperformed CRS for TTR prediction. More accurate, noninvasive, and early prediction of patient outcome may help reduce exposure to ineffective yet toxic chemotherapy or high-risk major hepatectomies.

## Introduction

Colorectal cancer is the second cause of cancer death in Canada and worldwide [1, 2]. Most patients progress by developing colorectal cancer liver metastases (CRLM). Resistance to systemic chemotherapy is the main determinant of patient survival and clinical decision making. While consensus is growing that it is unwise to continue the practice of treating large numbers of unselected patients who must endure treatment-related morbidities knowing only a fraction will benefit, there are currently few tools to reliably predict outcome after curative-indented treatments [3].

Better prediction tools would help identify subsets of patients at lowest risk of recurrence most likely to benefit from hepatectomy alone. In contrast, CRLM patients not achieving response with standard systemic chemotherapy could be offered non-surgical liver-directed therapy such as hepatic arterial infusion chemotherapy [4, 5]. Earlier assessment of CRLM response to chemotherapy would limit surgical morbidities related to hepatotoxicity, reduce unnecessary adverse events and cost in non-responding patients, and promote earlier therapeutic change with the opportunity to improve patient outcomes. In patients with operable CRLMs, the best validated pre-operative clinical risk score, which includes five clinicopathologic factors, cannot well identify patients with particularly good recurrence free-survival, and does not take into account the impact of chemotherapy [6, 7]. Fong’s clinical risk score (CRS), first proposed in [8] is commonly used in oncology for patient selection of candidates for surgical resection of CRLM and prediction of tumor recurrence.

Preoperative assessment of CRLM therapeutic response relies on visual assessment of follow-up computed tomography (CT) examinations by radiologists. However, interpretation is often limited by changes in CRLM appearance. Over the last decade, radiomics and deep learning approaches have been increasingly used in radiology [9–11] tackling various computer vision tasks such as detection [12, 13], classification, segmentation [14] but also in the context of survival analysis [15, 16]. Radiomics aim to leverage information embedded in medical images [17] by quantifying features related to textures, patterns or statistical parameters. Imaging features exhibited by liver metastases have been shown to be related to treatment response [18], overall survival [19] or disease-free survival [20]. However, there is a need to assess the ability of radiomics to predict clinical outcomes of patients with CRLM and to determine if they can improve the prediction performance when compared to the CRS, either alone or in combination.

The purpose of this study was to determine and compare the performance of pre-treatment CRS, radiomics models based on computed (CT) examinations, and their combination for predicting time to recurrence (TTR) and disease-specific survival (DSS) in patients with CRLM. More specifically, we used random survival forest (RSF) survival models and neural networks survival models (DeepSurv) to predict TTR and DSS. We then used radiomics signatures and DeepSurv scores, alone or in combination with CRS, to predict TTR and DSS as classification tasks. Performances of various tumor aggregations on outcome predictions are also presented.

## Materials and methods

### Study design and patients

All patient provided written consent to participate to the *Centre hospitalier de l’Université de Montréal* hepatopancreatobiliary cancer biobank and prospective registry, registered by the Canadian Tumour Repository Network [21], approved by the institutional ethics board (IRB) (No. 09.237, 22-Jan-2010), by which they accepted that clinical, radiological, and histo-pathological data be collected and used for research in specific projects further approved by the IRB. The current retrospective research project was approved on 31-May-2018 (IRB No. 18.023).

Patients with upfront resectable CRLM determined by treating oncologists and surgeons, treated between 2007 and 2017, who underwent a CT-scan within 3 months before and 10 days after beginning of pre-operative chemotherapy regimen, were selected.

**Fig 1:**
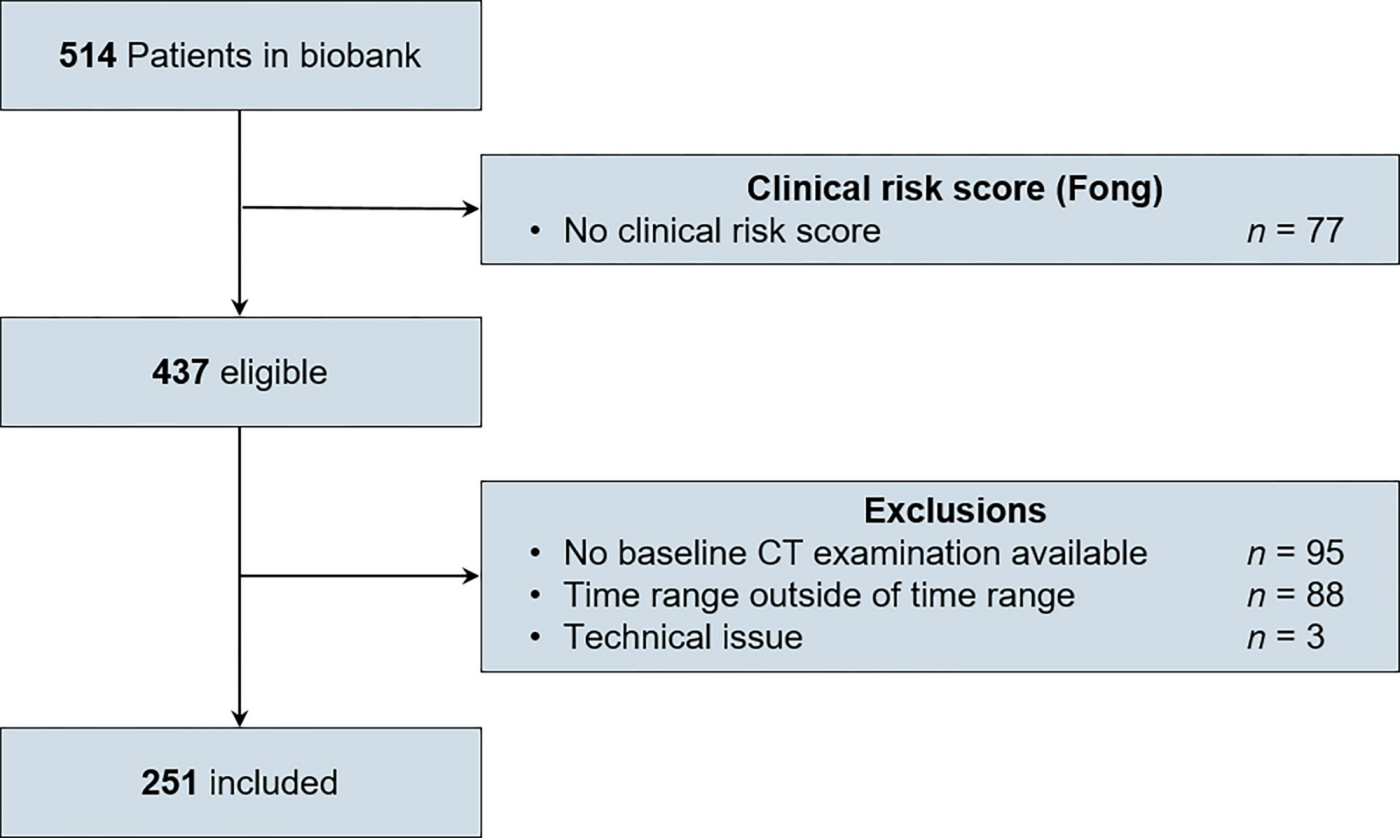
Flowchart of patient selection. Patients were selected from a prospectively maintained registry based on their imaging and treatment protocols. CT = computed tomography.

Flowchart of patient selection is presented in **Fig 1**.

### Clinical risk score

Based on clinical preoperative data, a clinical risk score (CRS) [22] ranging from 0 to 5 was calculated for each patient adding one point if the following features were present: node positive primary cancer, disease-free interval from primary to CRLM diagnosis of < 12 months, more than one CRLM, largest CRLM > 5 cm, and pre-operative carcinoembryonic antigen level > 200 ng/mL. The CRS was dichotomized as low risk (0-1-2) vs. high risk (3-4-5), as published [8].

### Imaging data

#### CT dataset

**Fig 2:**
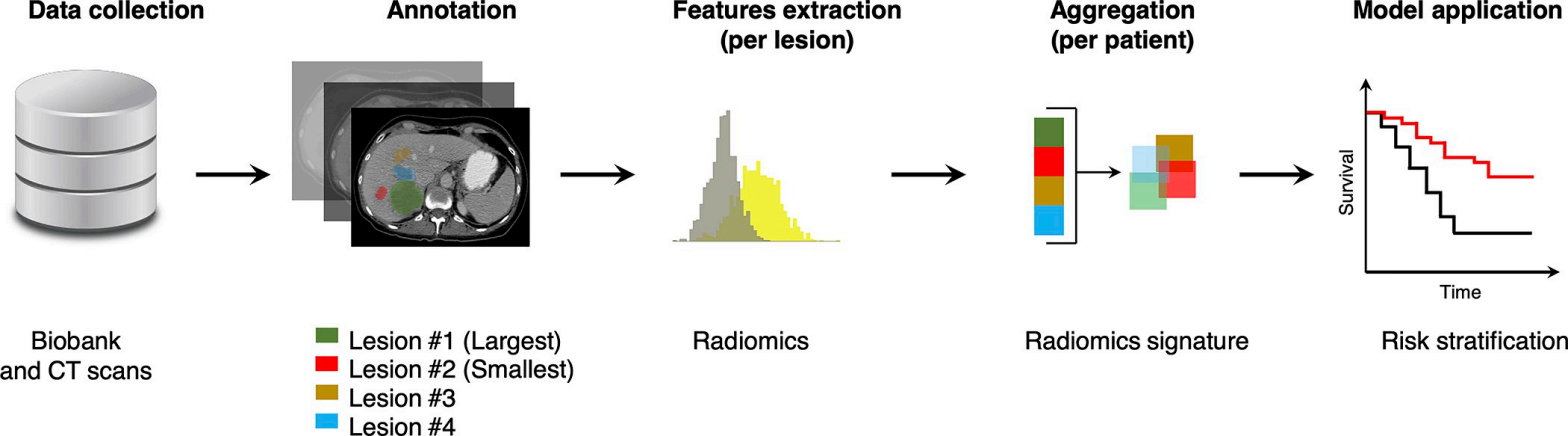
Data workflow. Patients from our prospectively maintained registry were selected based on their imaging and treatment protocols. CT exams were collected. All liver metastases were manually segmented. For each metastasis, 3-D radiomics were extracted and indexed, allowing multiple aggregation strategies. The radiomics signatures were used to stratify the risk of tumor recurrence and patient survival. CT = computed tomography.

For each patient, imaging data consisted in contrast-enhanced computed tomography (CT) pre-treatment examinations performed either before or within then days of first round of chemotherapy. CT scans were performed with various 16- and 64-multidetector scanners at our institution. All CT examinations included at least one portal venous phase covering the upper abdomen. CT scans acquired over a ten-year range (2007–2017) were retrospectively retrieved for research purposes between January 1, 2018 and June 30, 2018. Since patients were enrolled in a biobank, the authors had access to information that could identify individual participants during or after data collection. CT scans were performed on scanners from different manufacturers (Philips, Toshiba, GE, Siemens), models, variable Mas, and reconstruction kernels. The range of acquisition parameters are provided in **S1 Table**. Overall data workflow is presented in **Fig 2**.

#### Tumor segmentation

A total of 731 liver metastases have been segmented manually on CT images by 3 medical students. The segmentation masks were then reviewed and corrected, if needed, by a fellowship-trained radiologist. These masks were used to extract the radiomics features from liver metastases on CT examinations. The segmentation process was done using a free open-source image postprocessing software (The Medical Imaging Interaction Toolkit [MITK]; Heidelberg, Germany) [23]. Binary segmentation masks were saved for each CRLM.

#### Radiomics features computation

All images and masks have been resampled to 1 x 1 x 1 mm^3^ using the B-spline interpolation method to maintain a constant spatial sampling. Images were clipped between -100 and 200 Hounsfield units (HU) and bin width was set to 25. Masks and images were cropped one millimeter around lesion contours. Radiomics features from 3D lesions and masks were then extracted using PyRadiomics library [24]. Extracted features included first order, shape, gray level co-occurrence matrix (GLCM), gray level run length matrix (GLRLM), gray level size zone matrix (GLSZM), neighbouring gray tone difference matrix (NGTDM), gray level dependence matrix (GLDM) applied on original image, Laplacian of Gaussian filtered images with sigma ranging from 1 to 5 mm, and wavelet transformed images.

#### Radiomics features aggregation

**Fig 3:**
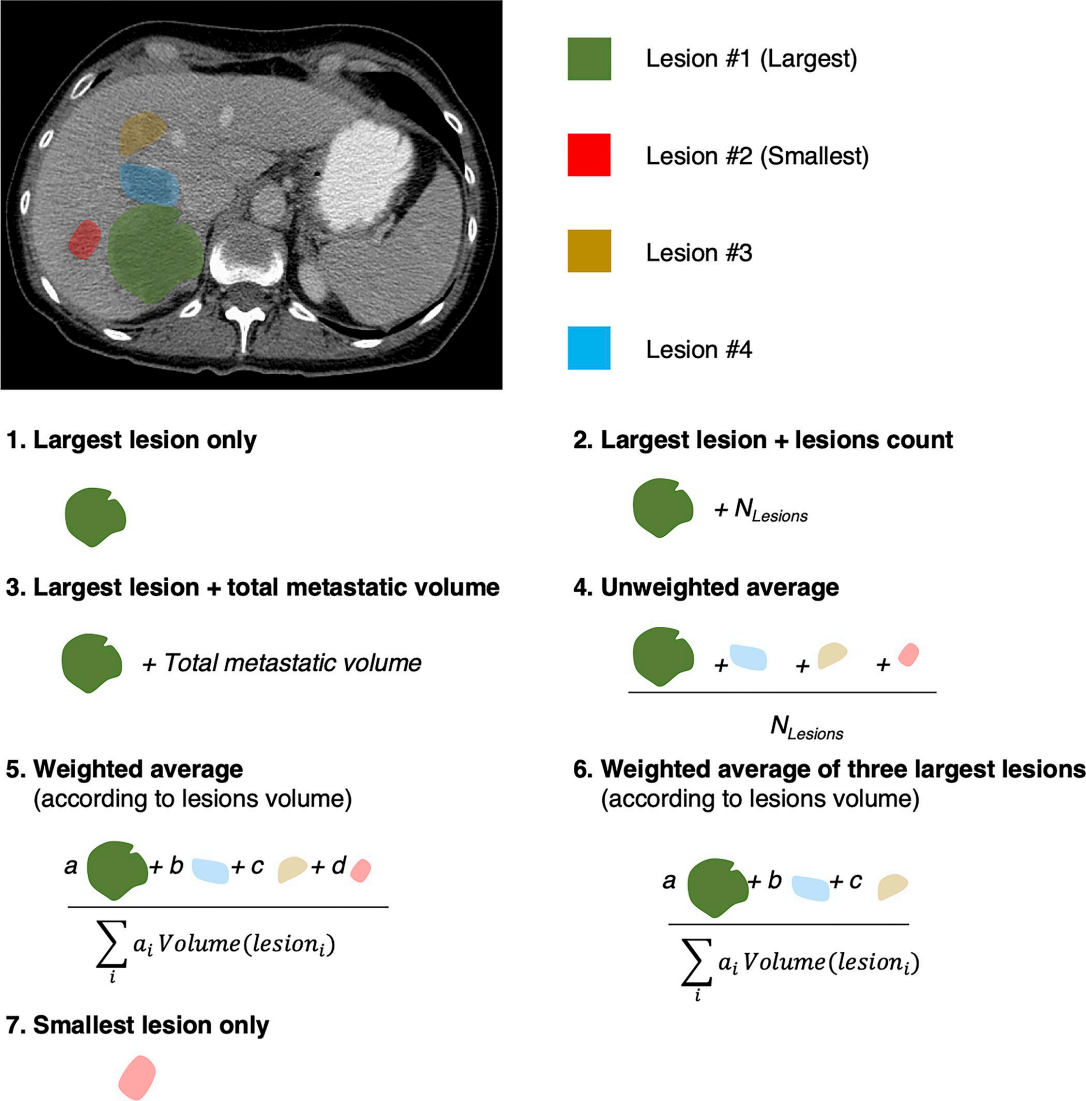
Investigated aggregation strategies. Considering multiple metastases, per lesion radiomic features are combined in six distinct schemes. Coefficients *a*,*b*,*c* and *d* indicate lesions volume ratios.

For each segmented metastasis, a 1-D array of 1,317 radiomics features is obtained. Thus, for a patient exhibiting *m* metastases a 2-D array of (*m x 1*,*317)* size is obtained. Since patients may present various amount of metastases, radiomic features extracted at the lesion scale need to be aggregated in order to leverage each lesion information and to retrieve features at patient scale, aiming further prediction (*e*.*g*., disease free survival, recurrence). Multiple aggregation strategies have been proposed in [25]. Investigated aggregation strategies are presented in **Fig 3**. For comparison, we also considered the smallest lesion.

For all strategies involving multiple lesions per patient, geometric features were summed while texture related features were averaged, as proposed in [25]. Thus, for each aggregation strategy, a unique radiomics dataset was obtained.

### Ground truth

Time to recurrence (TTR) was defined as the delay between date of curative surgery with complete CRLM resection and first diagnosis of tumor recurrence. Disease-specific survival (DSS) was defined as the delay between date of curative surgery with complete CRLM resection and cancer-related death. Patients who did not experience recurrence or cancer-related death were censored, with a time of observation set as the last follow up date.

### Dataset composition

For each TTR and DSS studies, a holdout test dataset representing 15% of the overall dataset was extracted, ensuring a similar ratio of events in both training and test datasets. *Radiomics signature*.*—*In order to reduce the number of features and to promote learning process, we first applied minimum redundancy maximum relevancy (MRMR) algorithm [26] on training datasets for both TTR and DSS studies, through its R language implementation [27], to extract the 50 first ranked features. Secondly, we used univariate Cox models to further reduce the number of features, keeping only features exhibiting a concordance index greater than 0.53 on the training dataset. Radiomics signature, which consists in a linear combination of radiomic features was established using a Cox proportional model using Lasso method as feature reduction method. Non-zero features and their associated coefficients were indexed to compute radiomics signature for each aggregation strategies. To compute the statistical distribution of radiomics features, models were trained 100 times on a random subset representing 85% of the training dataset. Such approach avoids data leaking by excluding the test dataset in the feature selection step, while allowing bootstrapping.

### Recurrence and survival prediction

#### Models

We considered two distinct survival models, namely random survival forest (RSF) [28] from the *Scikit-Surv* package [29], and neural network survival models derived from DeepSurv architecture proposed in [30]. Backbone model consisted in two consecutive blocks, each consisting in a 4-node fully connected layers, batch normalization, and a 0.1 coefficient dropout layer. Combination of radiomic features with CRS consisted in concatenating CRS to selected features in survival models training datasets.

For each model, training was performed 100 times on distinct random splits of the train dataset at a 85% ratio. Metrics on holdout dataset were indexed, providing statistics for survival analysis and classification tasks. Survival models were trained using either radiomic only or in combination with CRS. Classification step consisted in applying logistic regression to survival radiomics signatures scores alone or combined with CRS (1 or 2 features). Same approach was used for DeepSurv scores. To avoid overfitting, neural network survival models training was stopped at the lowest validation dataset loss, which consisted in the 15% remaining from the train dataset split. Adam optimizer with a 0.00001 learning rate was used for training. In order to address radiomic signatures relevance, standard classification from selected radiomic features is investigated for both TTR and DSS prediction using logistic regression and random forest classifiers.

### Statistical analysis

Concordance index, which is a common evaluation metric of survival models, relies on correlation between predicted score and observed events. Concordance index was computed for CRS alone, radiomics signature alone, and the combination of both. Area under the curve (AUC) was computed from receiver operating characteristic curves and used as classification metric. To compute the AUC, the dichotomization was based on the time interval that provided balanced groups, which was recurrence status at 18 months for TTR and survival at 3 years for DSS.

Confidence intervals were computed using bootstrapping method over two thousands iterations.

## Results

### Study database

**Table 1** summarizes characteristics of patients, tumors, clinical risk score, and chemotherapy received. The majority of patients were males (64.3%). The mean age of patients was 62.9 ± 9.5 years. The size of metastases ranged from 5 to 205 mm. The clinical risk score was distributed as follows: 7, 42, 93, 84, 14, and 1 patients had Fong’s scores of 0 to 5, respectively. All patients (241/241) received chemotherapy before surgery and a majority of patients (202/241) also received chemotherapy after surgery.

**Table 1:** Clinical characteristics of 241 patients.

Characteristics	Data
**Patients**	
Sex	
Male	155/241 (64.3)
Female	86/241 (35.7)
Age (years) ± SD	62.9 ± 9.5
Number of liver metastases before chemotherapy	3.0 ± 3.0
**Tumors**	
Metastases size (mm)	
Mean ± SD	32.7 ± 22.9
Range	5.3–204.6
**Clinical risk score**	
Fong’s score	
0	7/241 (2.9)
1	42/241 (17.4)
2	93/241 (38.6)
3	84/241 (34.9)
4	14/241 (5.8)
5	1/241 (0.4)
**Chemotherapy**	
Received chemotherapy before surgery	241/241 (100)
First chemotherapy duration (days)	66.0 ± 50.0
Folfox-based	217/241 (90.0)
5-fluorouracil-based combination	239/241 (99.2)
Received chemotherapy after surgery	202/241 (85.9)
First post-surgery chemotherapy duration (days)	91.8 ± 55.6
Folfox-based	155/202 (76.7)
5-fluorouracil-based combination	187/202 (92.6)

Numbers in parentheses are percentages. SD = standard deviation.

### Time to recurrence prediction

**Fig 4:**
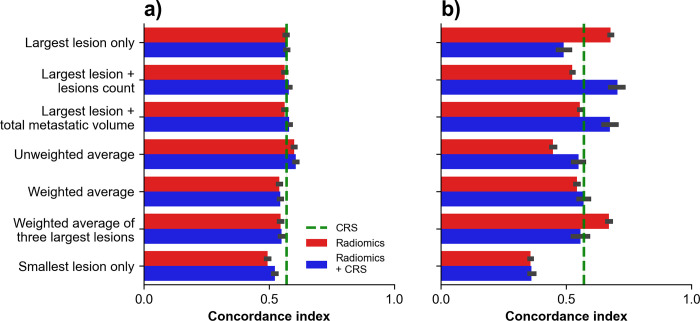
Time to recurrence (TTR) prediction using survival models. Concordance indexes obtained on holdout test dataset using survival models across aggregation strategies for TTR prediction with (A) random survival forests and (B) DeepSurv. Green dashed line indicates performance of CRS alone.

Bar plots of concordance indexes obtained for TTR prediction using survival models across aggregation strategies are shown in **Fig 4**. For RSF, the highest performance was obtained with ‘unweighted average’ in combination with CRS which provided a concordance index of 0.61 (95% confidence interval: 0.60–0.61). For DeepSurv-44 the highest performance was obtained with ‘largest lesion + lesions count’ aggregation in combination with CRS which provided a concordance index of 0.70 (0.68–0.73).

### Disease-specific survival prediction

**Fig 5:**
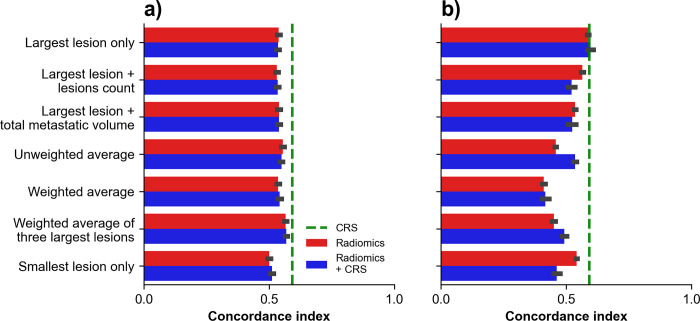
Disease-specific survival (DSS) prediction using survival models. Concordance indexes obtained on holdout test dataset using survival models across aggregation strategies for DSS prediction with (A) random survival forests and (B) DeepSurv. Green dashed line indicates performances of CRS alone.

Bar plots of concordance indexes obtained for DSS prediction using survival models across aggregation strategies are shown in **Fig 5**. For RSF, the highest performance was obtained with the aggregation ‘weighted average of three largest lesions’ which provided a concordance index of 0.57 (0.56–0.57). For DeepSurv-44 the highest performance was obtained with the aggregation ‘largest lesion only’ which provided a concordance index of 0.60 (0.58–0.61).

### Time to recurrence classification

**Fig 6:**
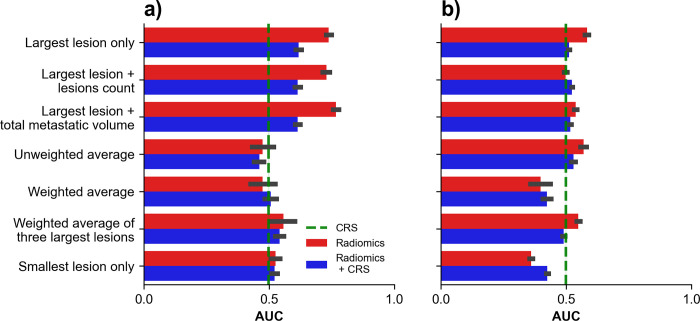
Time to recurrence (TTR) classification using radiomics signatures. AUC obtained on holdout test dataset using radiomics signatures across aggregation strategies for TTR classification with (A) radiomics signature and (B) DeepSurv score. Green dashed line indicates performances of CRS alone.

Bar plots of AUCs obtained for TTR classification using radiomic signatures across aggregation strategies are shown in **Fig 6**. For radiomics signature, the highest performance was obtained with the aggregation ‘largest and total metastatic volume’ providing an AUC of 0.77 (0.75–0.78). For DeepSurv-44, the highest performance was obtained with aggregation ‘Largest lesion only’ providing an AUC of 0.58 (0.57–0.59).

Bar plots of AUCs obtained for TTR classification using selected radiomic features across aggregation strategies are shown in **S1 Fig.** For logistic regression, the highest performance was obtained with the aggregation ‘smallest’ combine with CRS providing an AUC of 0.40 (0.39–0.41). For random forest, the highest performance was obtained with aggregation ‘Unweighted average’ combined with CRS, providing an AUC of 0.50 (0.48–0.51).

### Disease-specific survival classification

**Fig 7:**
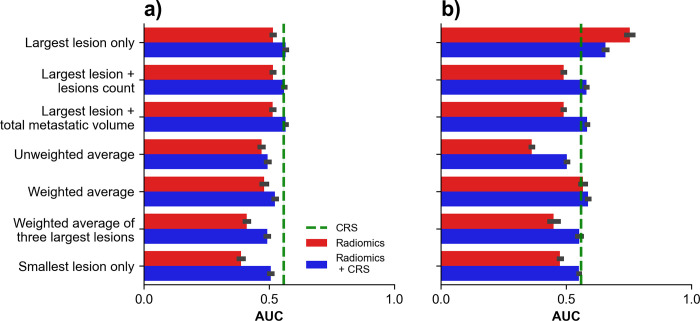
Disease-specific survival (DSS) classification using radiomics signatures. AUC obtained on holdout test dataset using radiomics signatures across aggregation strategies for DSS classification with (A) radiomics signature and (B) DeepSurv score. Green dashed line indicates performances of CRS alone.

Bar plots of AUCs obtained for disease-specific survival classification using radiomic signatures across aggregation strategies are shown in **Fig 7**. For radiomics signature, the highest performance was obtained with the aggregation ‘largest lesion only’ combined with CRS which provided an AUC of 0.57 (0.56–0.57). For DeepSurv, the highest performance was obtained with the aggregation ‘largest lesion only’ which provided an AUC of 0.75 (0.74–0.76).

Bar plots of AUCs obtained for disease-specific survival classification using selected radiomic signatures across aggregation strategies are shown in **S2 Fig**. For logistic regression, the highest performance was obtained with the aggregation ‘weighted average of the three largest lesions’ which provided an AUC of 0.55 (0.54–0.57). For random forest, the highest performance was obtained with the aggregation ‘weighted average’ combined with CRS which provided an AUC of 0.63 (0.62–0.65).

### Radiomics signature coefficients

**Fig 8:**
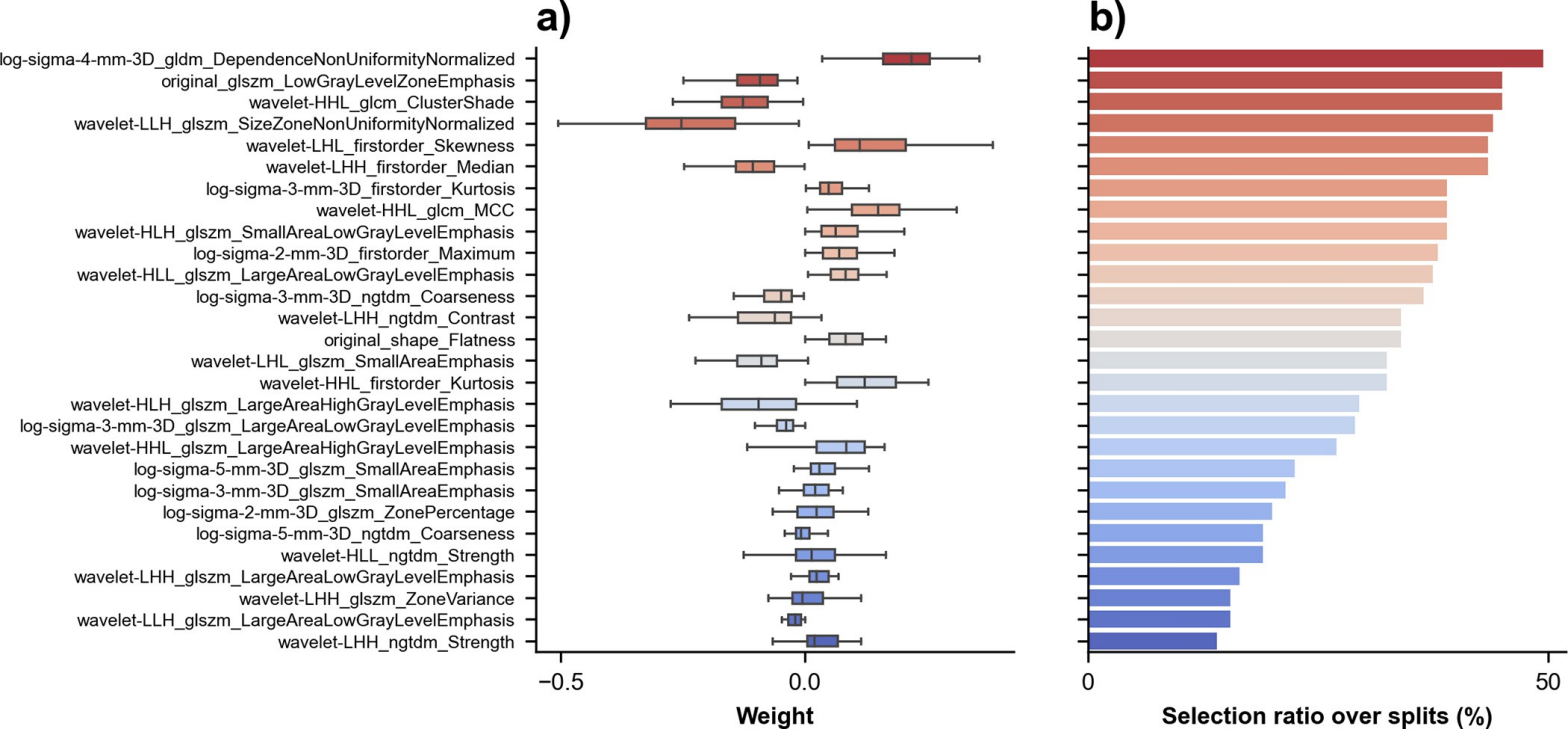
Radiomics signature coefficients for prediction of recurrence. (A) Box plot shows selected features coefficients sorted by descending order of selection ratio over splits. (B) Count plot of radiomics signature coefficients selections over the splits for the aggregation the radiomics signature coefficients with the aggregation ‘largest lesion only’.

Box plot depicting variability of selected features coefficients and count plot of radiomics signature coefficients selections over the splits are shown in **Fig 8** for ‘Largest lesion only’ in TTR study. A majority of features were selected in about half of the splits depicting their contribution to the final radiomics signature. Similar plot for DSS study is provided in **S3 Fig**. Initial sets of selected features for ‘Largest lesion only’ are provided in **S2 Table**. Dataset is provided in **S1 File**.

**S3 Table** indexes selected features for DeepSurv-44 training per aggregation type.

**Fig 9:**
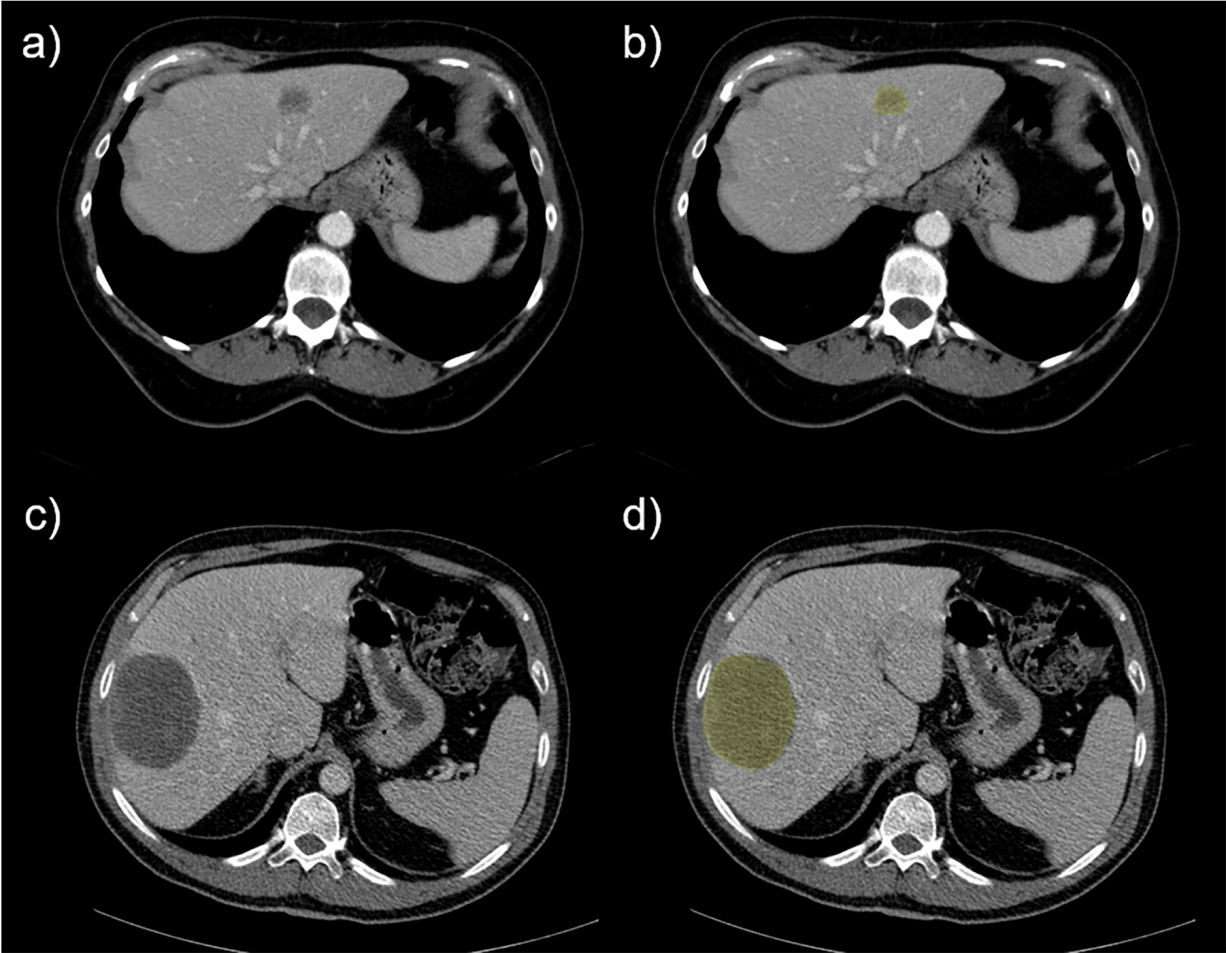
Liver metastases examples with their associated segmentations. First row exhibits (A) initial CT image (left) and (B) a segmented metastasis (yellow area) of the patient with the shortest survival in our dataset. Second row exhibits (C) initial CT image (left) and (D) a segmented metastasis (yellow) of the patient with the longest survival in our dataset.

**Fig 9** shows representative examples of patients with shortest and longest survival times.

## Discussion

In this retrospective study derived from a prospective registry, we evaluated the use of radiomics features and signatures on pre-treatment, baseline CT images, to predict recurrence and survival after complete resection of CRLM. We initially identified radiomics features providing the highest prediction performance using dedicated feature selection methods. We compared different feature aggregation strategies to account for the presence of multiple CRLM in patients.

Demircioğlu A et al. [31] has shown that performing feature selection before cross-validation may induce data leakage and bias results. In this study, radiomics features were selected on the training dataset only, thus avoiding any data leakage from the holdout test dataset. We considered linear combinations of radiomics features at the patient level (aggregation of multiple radiomics features across lesions) at each training iteration, over the 100 training dataset splits. We then compared survival models based on CRS, radiomics signatures, and their combinations. A multivariate analysis including neoadjuvant chemotherapy characteristics and histological results has not been performed due to the wide range of treatment strategies. We focused on imaging and clinical data available at baseline, aiming for early prediction of oncological outcomes.

Because patients often have multiple CRLM, we had to investigate various aggregation strategies. ‘Largest lesion only’ aggregation strategy and its derivatives ranked first in both TTR and DSS classification tasks, regardless of the predictor used (radiomics signature or DeepSurv score). This finding is in agreement with the common practice of using the largest tumor among index lesions followed over time for the assessment of treatment response and also with the concept of tumor burden, a well known prognostic factor [32]. Conversely, ‘Smallest lesion only’ aggregation strategy ranked last in most scenarios, further reinforcing the finding that larger observations have a higher predictive value. Average-based aggregation strategies led to poor-to-moderate performance in almost all cases, except processed by neural networks in some cases. Averaging may induce a loss of characteristic imaging information, especially regarding texture-based features, as large metastases exhibit different textures compared to small ones.

Some recent studies have examined radiomics features to predict treatment response. Lubner et al. has performed CT texture analysis of untreated CRLM and found that different radiomics features predicted pathological response to preoperative chemotherapy and clinical outcomes [33]. Rao et al. previously examined radiomics features such as relative differences in CT texture occurring after treatment of CRLM and found that Δuniformity and Δentropy were better for predicting response to chemotherapy than changes in lesion size or volume [18]. Froelich et al. found that increased attenuation of untreated CRLM constituted a prognostic factor of prolonged overall survival [34]. Considering features from ‘Largest lesion only’ survival radiomics signature presented in **Fig 8**, one can note the importance of wavelet-filtered image radiomics and the recurrence of features derived from gray level emphasis. Such trend is aligned with observations from Mostafavi et al. [35] regarding treatment response. Furthermore, like Ravanelli et al. [36] and Simpson et al. [37] who highlighted the link between density and correlation with overall survival, we observe three density related features and two correlation related features in our DSS radiomic signature shown in **S3 Fig**.

Including the CRS in radiomics-based models lead to improvements in prediction performance in only a few specific configurations, such as TTR prediction. However, CRS alone provided good concordance indexes despite its apparent simplicity. In this context, CRS or other prognostic system [38] may be used in future studies as a basis for comparison or in a multi-omics context.

The proposed neural network survival models outperformed RSF in our dataset. Since RSF are prone to overfitting, fine-tuned architectures may be required to increase performances, such as reducing the number of classifiers, or maximum depth. This would be consistent with the light neural network architecture used in study, which showed better generalization on test dataset in most of investigated configurations.

In the classification task of TTR and DSS, both logistic regression and random survival forest applied to selected radiomics features provided lower AUCs than radiomics signatures scores, combined or not with CRS, thus confirming the relevance of the proposed approach.

To our knowledge, this is the first study comparing combination of radiomics signatures and clinical risk score across multiple aggregation strategies through RSF and survival network models and validated on a holdout test dataset to provide insights on clinical outcomes in patients with CRLM.

### Limitations

External and prospective validation on independent datasets should be pursued for validation of our findings. Technical parameters such as slice thickness, reconstruction kernel, and scanner manufacturer are known to be sources of variability affecting the statistical distribution of radiomic features [39]. Radiomics reproducibility and robustness remains a current active topic in machine learning applied to medicine [40–42]. Since the objective of this work was to predict outcomes on baseline CT, we did not evaluate use the Response Evaluation Criteria In Solid Tumors (RECIST), which is also not used to guide surgical decision in patients with resectable CRLM in practice.

## Conclusions

In conclusion, radiomics features using baseline CT provided a good accuracy for predicting recurrence and disease-specific survival in patients with CRLM undergoing chemotherapy and complete resection. These results await validation of these imaging-based prognostic biomarkers in larger cohort to guide the design of future prospective studies.

## Supporting information

S1 Fig Time to recurrence (TTR) classification using selected radiomic features. AUC obtained on holdout test dataset using radiomics features across aggregation strategies for TTR classification using (A) logistic regression and (B) random forest. Gray dashed line indicates randomness.
(TIFF)

S2 Fig Disease-specific survival (DSS) classification using selected radiomic features. AUC obtained on holdout test dataset using radiomics features across aggregation strategies for DSS classification using (A) logistic regression and (B) random forest. Gray dashed line indicates randomness.
(TIFF)

S3 Fig Radiomics signature coefficients for prediction of survival. (A) Box plot shows selected features coefficients sorted by descending order of selection ratio over splits. (B) Count plot of radiomics signature coefficients selections over the splits for the aggregation the radiomics signature coefficients with the aggregation ‘largest lesion only’.
(TIFF)

S1 Table Scanners and imaging parameters characteristics. (DOCX)

S2 Table Radiomic features selected prediction of recurrence and survival for ‘Largest lesion only’ aggregation. Radiomic features selected using ‘Largest lesion only’ aggregation for recurrence and survival predictions, and the intersection of the two features sets.
(DOCX)

S3 Table Selected radiomic features for DeepSurv-44 per aggregation type. (DOCX)

S1 File Radiomics datasets per aggregation type. (ZIP)

S4 CRLM Dataset (ZIP)

## Abbreviations

CI: concordance index
CRLM: colorectal cancer liver metastases
CRS: Clinical risk score
CT: computed tomography
DSS: disease-specific survival
GLCM: gray level co-occurrence matrix
GLDM: gray level dependence matrix
GLRLM: gray level run length matrix
GLSZM: gray level size zone matrix
MRMR: minimum redundancy maximum relevancy
NGTDM: neighbouring gray tone difference matrix
RECIST: response evaluation criteria in solid tumors
RSF: random survival forest
TTR: time to recurrence
