# Supplementary figures and images for: Radiomics analysis of baseline computed tomography to predict oncological outcomes in patients treated for resectable colorectal cancer liver metastasis
Source: PLoS One. 2024 Sep 11;19(9):e0307815. doi: 10.1371/journal.pone.0307815 (PMC11389941; doi:10.1371/journal.pone.0307815)

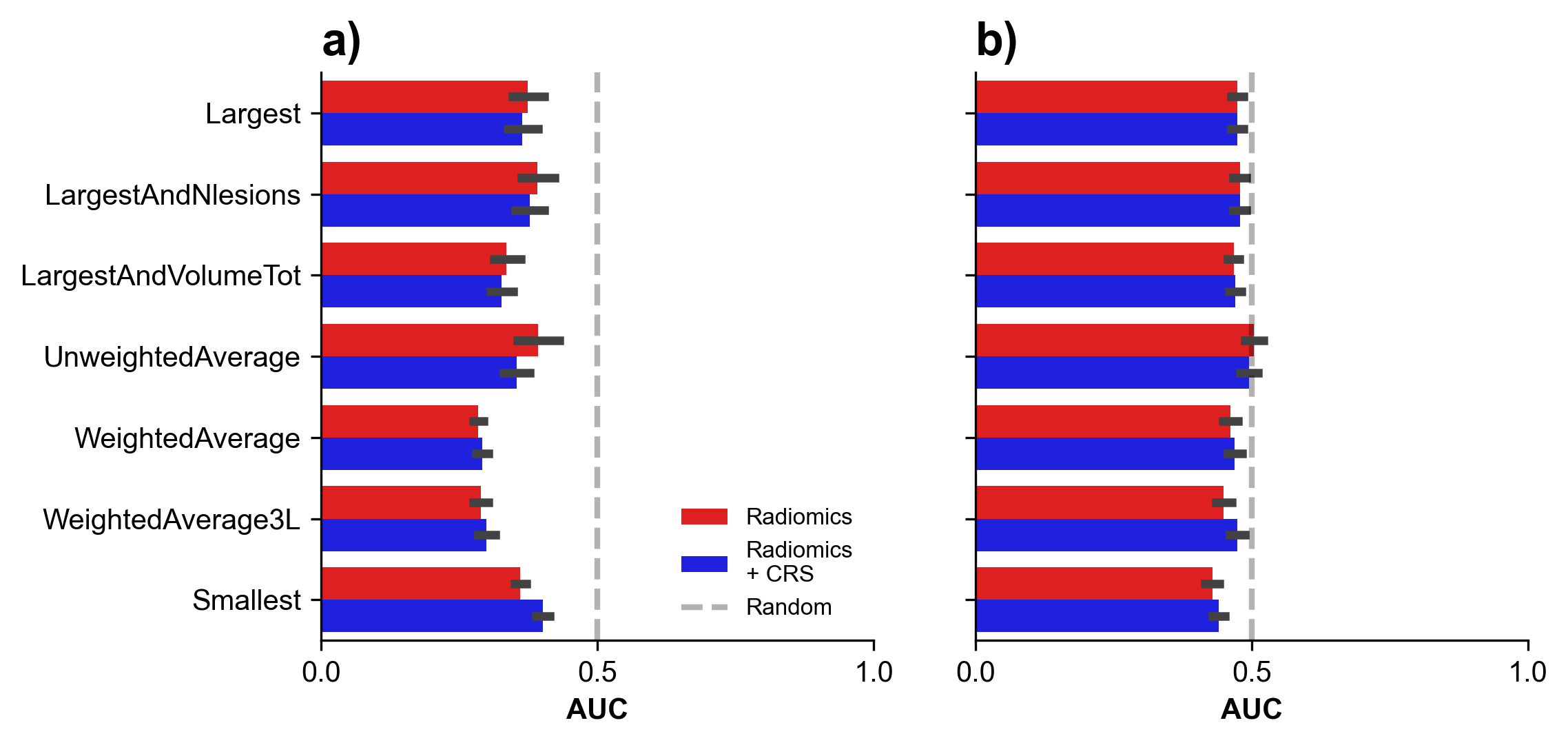

Supplement: S1 Fig — AUC obtained on holdout test dataset using radiomics features across aggregation strategies for TTR classification using (A) logistic regression and (B) random forest. Gray dashed line indicates randomness. (TIFF) [file pone.0307815.s001.tiff]

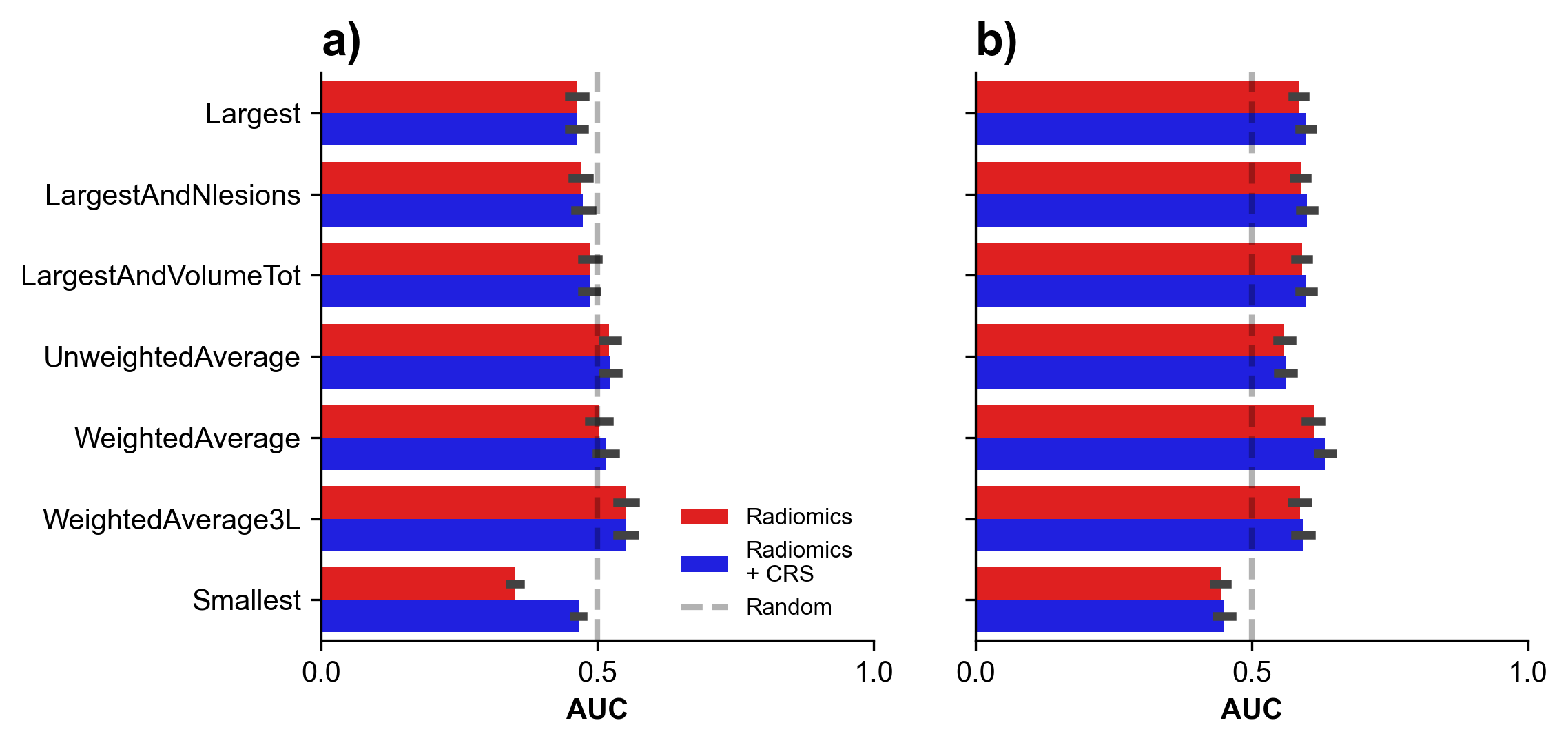

Supplement: S2 Fig — AUC obtained on holdout test dataset using radiomics features across aggregation strategies for DSS classification using (A) logistic regression and (B) random forest. Gray dashed line indicates randomness. (TIFF) [file pone.0307815.s002.tiff]

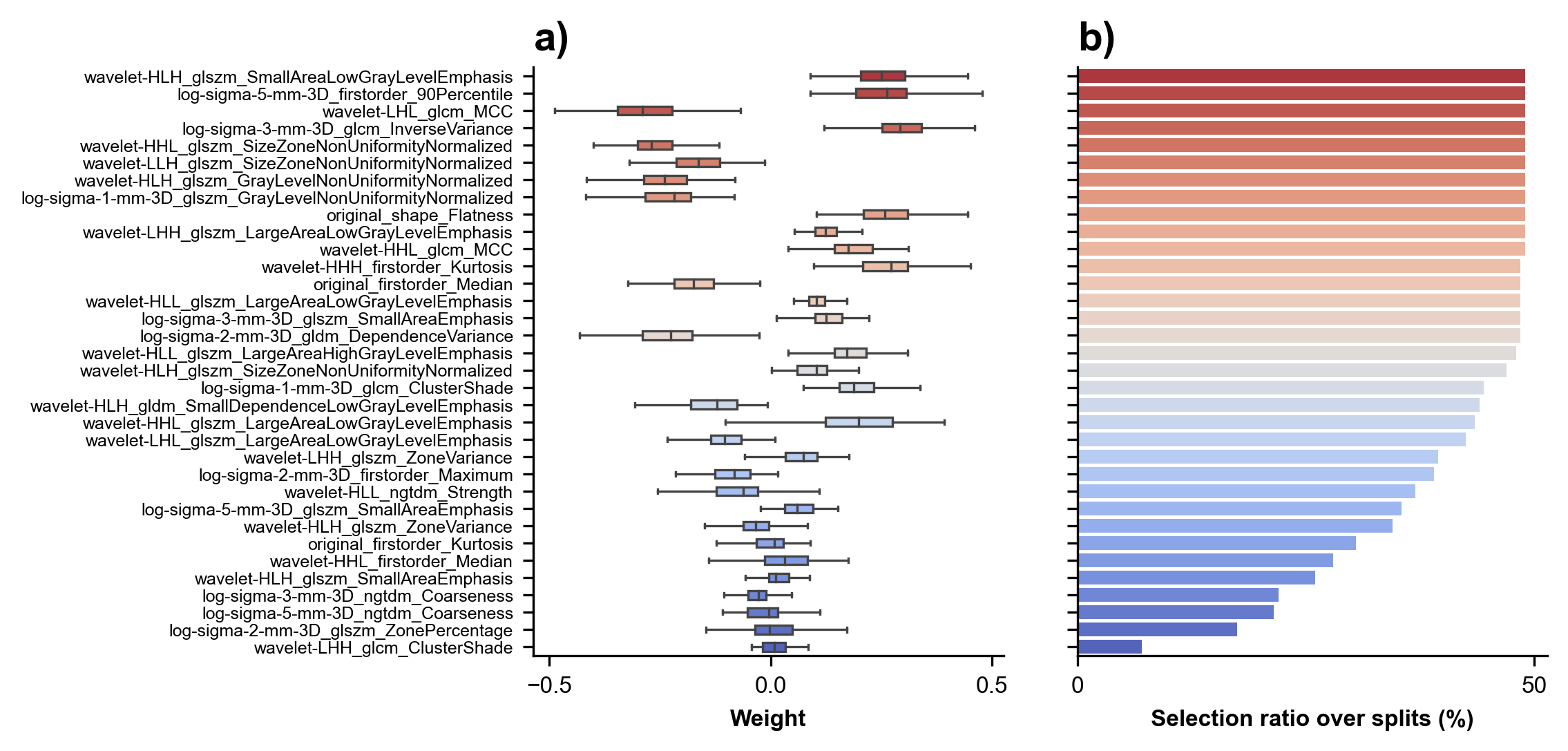

Supplement: S3 Fig — (A) Box plot shows selected features coefficients sorted by descending order of selection ratio over splits. (B) Count plot of radiomics signature coefficients selections over the splits for the aggregation the radiomics signature coefficients with the aggregation ‘largest lesion only’. (TIFF) [file pone.0307815.s003.tiff]
