# Supplementary material for: Radiomics analysis of baseline computed tomography to predict oncological outcomes in patients treated for resectable colorectal cancer liver metastasis
Source: PLoS One. 2024 Sep 11;19(9):e0307815. doi: 10.1371/journal.pone.0307815 (PMC11389941; doi:10.1371/journal.pone.0307815)
Supplement: S1 Table — (DOCX) [file pone.0307815.s004.docx]

**S1 Table.** Scanners and imaging parameters characteristics.

| **Scanners** | |
| --- | --- |
| **Philips** |  |
| Model | Brilliance 64 |
| Kernel | B |
| **General Electric** |  |
| Models | BrightSpeed, LightSpeed16, OptimaCT660 |
| Kernels | Standard |
| **Toshiba** |  |
| Model | Aquilion |
| Kernels | FC02, FC14, FC17 |
| **Siemens** |  |
| Models | Somatom, Sensation, Definition |
| Kernels | B40f, B25f |
| **Imaging parameters** | |
| Slice spacing (mm) | 3.0 ± 1.3 |
| Slice thickness (mm) | 3.47 ± 1.27 |
| Exposure time (ms) | 799.6 ± 194.6 |
| Dose-length product (mGy.cm) | 405.4 ± 234.8 |
| CT beam energy (kV) | 119.15 ± 5.8 |
| X-ray tube current (mAs) | 190.7 ± 110.0 |
| Spiral pitch factor | 1.18 ± 0.31 |
| Scan length (mm) | 422.2 ± 149.6 |
|  |  |
