# Supplementary material for: Radiomics analysis of baseline computed tomography to predict oncological outcomes in patients treated for resectable colorectal cancer liver metastasis
Source: PLoS One. 2024 Sep 11;19(9):e0307815. doi: 10.1371/journal.pone.0307815 (PMC11389941; doi:10.1371/journal.pone.0307815)
Supplement: S2 Table — Radiomic features selected using ‘Largest lesion only’ aggregation for recurrence and survival predictions, and the intersection of the two features sets. (DOCX) [file pone.0307815.s005.docx]

**S2 Table.** **Selected Radiomic Features**. Radiomic features selected using ‘Largest lesion only’ aggregation for recurrence and survival predictions, and the intersection of the two features sets.

| Recurrence | [log-sigma-2-mm-3D_glszm_ZonePercentage, log-sigma-4-mm-3D_gldm_DependenceNonUniformityNormalized, wavelet-LHH_glszm_ZoneVariance, log-sigma-2-mm-3D_firstorder_Maximum, wavelet-HHL_glcm_ClusterShade, log-sigma-3-mm-3D_glszm_LargeAreaLowGrayLevelEmphasis, log-sigma-3-mm-3D_ngtdm_Coarseness, wavelet-HLH_glszm_LargeAreaHighGrayLevelEmphasis, wavelet-LHH_ngtdm_Strength, wavelet-HLL_glszm_LargeAreaLowGrayLevelEmphasis, wavelet-HLL_ngtdm_Strength, original_glszm_LowGrayLevelZoneEmphasis, log-sigma-3-mm-3D_firstorder_Kurtosis, wavelet-LHL_firstorder_Skewness, wavelet-LLH_glszm_LargeAreaLowGrayLevelEmphasis, wavelet-LHH_glszm_LargeAreaLowGrayLevelEmphasis, wavelet-LLH_glszm_SizeZoneNonUniformityNormalized, wavelet-HHL_glszm_LargeAreaHighGrayLevelEmphasis, wavelet-LHH_ngtdm_Contrast, log-sigma-5-mm-3D_glszm_SmallAreaEmphasis, log-sigma-3-mm-3D_glszm_SmallAreaEmphasis, wavelet-LHH_firstorder_Median, wavelet-HHL_firstorder_Kurtosis, wavelet-HLH_glszm_SmallAreaLowGrayLevelEmphasis, wavelet-HHL_glcm_MCC, wavelet-LHL_glszm_SmallAreaEmphasis, original_shape_Flatness, log-sigma-5-mm-3D_ngtdm_Coarseness] |
| --- | --- |
| Survival | [wavelet-HLH_glszm_SmallAreaEmphasis, wavelet-HLH_glszm_ZoneVariance, wavelet-HLH_glszm_SmallAreaLowGrayLevelEmphasis, wavelet-HLH_gldm_SmallDependenceLowGrayLevelEmphasis, wavelet-HLH_glszm_GrayLevelNonUniformityNormalized, log-sigma-2-mm-3D_glszm_ZonePercentage, wavelet-LHL_glszm_LargeAreaLowGrayLevelEmphasis, log-sigma-3-mm-3D_ngtdm_Coarseness, wavelet-HLH_glszm_SizeZoneNonUniformityNormalized, wavelet-LLH_glszm_SizeZoneNonUniformityNormalized, wavelet-LHH_glszm_ZoneVariance, log-sigma-2-mm-3D_firstorder_Maximum, log-sigma-1-mm-3D_glszm_GrayLevelNonUniformityNormalized, wavelet-HHL_glszm_LargeAreaLowGrayLevelEmphasis, log-sigma-3-mm-3D_glszm_SmallAreaEmphasis, wavelet-HLL_glszm_LargeAreaLowGrayLevelEmphasis, wavelet-HLL_ngtdm_Strength, log-sigma-2-mm-3D_gldm_DependenceVariance, log-sigma-1-mm-3D_glcm_ClusterShade, wavelet-HLL_glszm_LargeAreaHighGrayLevelEmphasis, log-sigma-5-mm-3D_firstorder_90Percentile, log-sigma-3-mm-3D_glcm_InverseVariance, wavelet-HHL_firstorder_Median, wavelet-LHL_glcm_MCC, wavelet-HHL_glszm_SizeZoneNonUniformityNormalized, log-sigma-5-mm-3D_ngtdm_Coarseness, wavelet-HHH_firstorder_Kurtosis, original_firstorder_Kurtosis, original_firstorder_Median, wavelet-LHH_glszm_LargeAreaLowGrayLevelEmphasis, wavelet-HHL_glcm_MCC, log-sigma-5-mm-3D_glszm_SmallAreaEmphasis, original_shape_Flatness, wavelet-LHH_glcm_ClusterShade] |
| Common features | [wavelet-LHH_glszm_LargeAreaLowGrayLevelEmphasis, log-sigma-2-mm-3D_glszm_ZonePercentage, wavelet-HHL_glcm_MCC, original_shape_Flatness, log-sigma-5-mm-3D_glszm_SmallAreaEmphasis, log-sigma-5-mm-3D_ngtdm_Coarseness, log-sigma-3-mm-3D_ngtdm_Coarseness, wavelet-HLH_glszm_SmallAreaLowGrayLevelEmphasis, log-sigma-2-mm-3D_firstorder_Maximum, wavelet-HLL_glszm_LargeAreaLowGrayLevelEmphasis, wavelet-LLH_glszm_SizeZoneNonUniformityNormalized, log-sigma-3-mm-3D_glszm_SmallAreaEmphasis, wavelet-HLL_ngtdm_Strength, wavelet-LHH_glszm_ZoneVariance] |
