# Supplementary material for: Radiomics analysis of baseline computed tomography to predict oncological outcomes in patients treated for resectable colorectal cancer liver metastasis
Source: PLoS One. 2024 Sep 11;19(9):e0307815. doi: 10.1371/journal.pone.0307815 (PMC11389941; doi:10.1371/journal.pone.0307815)
Supplement: S3 Table — (DOCX) [file pone.0307815.s006.docx]

**S3 Table.** **Selected Radiomic Features for DeepSurv-44 per aggregation type**. Radiomic features selected for recurrence and survival predictions, stratified by aggregation type.

| **Task** | **Aggregation type** | **Features** |
| --- | --- | --- |
| recurrence | Largest | ['log-sigma-4-mm-3D_gldm_DependenceNonUniformityNormalized', 'log-sigma-2-mm-3D_firstorder_Maximum', 'wavelet-HHL_glcm_ClusterShade', 'original_glszm_LowGrayLevelZoneEmphasis', 'log-sigma-3-mm-3D_firstorder_Kurtosis', 'wavelet-LLH_glszm_SizeZoneNonUniformityNormalized', 'wavelet-LHH_firstorder_Median', 'log-sigma-3-mm-3D_ngtdm_Coarseness', 'wavelet-HLL_glszm_LargeAreaLowGrayLevelEmphasis', 'wavelet-LHL_firstorder_Skewness', 'wavelet-LHH_ngtdm_Contrast', 'wavelet-HHL_firstorder_Kurtosis', 'wavelet-HLH_glszm_SmallAreaLowGrayLevelEmphasis', 'wavelet-HHL_glcm_MCC', 'original_shape_Flatness', 'log-sigma-2-mm-3D_glszm_ZonePercentage', 'log-sigma-3-mm-3D_glszm_LargeAreaLowGrayLevelEmphasis', 'wavelet-HLH_glszm_LargeAreaHighGrayLevelEmphasis', 'wavelet-LHH_glszm_LargeAreaLowGrayLevelEmphasis', 'wavelet-HHL_glszm_LargeAreaHighGrayLevelEmphasis', 'log-sigma-5-mm-3D_glszm_SmallAreaEmphasis', 'wavelet-LHL_glszm_SmallAreaEmphasis', 'log-sigma-3-mm-3D_glszm_SmallAreaEmphasis', 'wavelet-HLL_ngtdm_Strength', 'wavelet-LHH_glszm_ZoneVariance', 'wavelet-LHH_ngtdm_Strength', 'wavelet-LLH_glszm_LargeAreaLowGrayLevelEmphasis', 'log-sigma-5-mm-3D_ngtdm_Coarseness'] |
| recurrence | LargestAndNlesions | ['log-sigma-4-mm-3D_gldm_DependenceNonUniformityNormalized', 'log-sigma-2-mm-3D_firstorder_Maximum', 'wavelet-HHL_glcm_ClusterShade', 'original_glszm_LowGrayLevelZoneEmphasis', 'log-sigma-3-mm-3D_firstorder_Kurtosis', 'wavelet-LLH_glszm_SizeZoneNonUniformityNormalized', 'wavelet-LHH_firstorder_Median', 'log-sigma-2-mm-3D_glszm_ZonePercentage', 'wavelet-LHH_glszm_ZoneVariance', 'log-sigma-3-mm-3D_glszm_LargeAreaLowGrayLevelEmphasis', 'log-sigma-3-mm-3D_ngtdm_Coarseness', 'wavelet-HLH_glszm_LargeAreaHighGrayLevelEmphasis', 'wavelet-LHH_ngtdm_Strength', 'wavelet-HLL_glszm_LargeAreaLowGrayLevelEmphasis', 'wavelet-HLL_ngtdm_Strength', 'wavelet-LHL_firstorder_Skewness', 'wavelet-LHH_glszm_LargeAreaLowGrayLevelEmphasis', 'wavelet-HHL_glszm_LargeAreaHighGrayLevelEmphasis', 'wavelet-LHH_ngtdm_Contrast', 'log-sigma-3-mm-3D_glszm_SmallAreaEmphasis', 'wavelet-HHL_firstorder_Kurtosis', 'wavelet-HLH_glszm_SmallAreaLowGrayLevelEmphasis', 'wavelet-HHL_glcm_MCC', 'wavelet-LHL_glszm_SmallAreaEmphasis', 'original_shape_Flatness', 'Nlesions', 'log-sigma-5-mm-3D_glszm_SmallAreaEmphasis', 'wavelet-LLH_glszm_LargeAreaLowGrayLevelEmphasis', 'log-sigma-5-mm-3D_ngtdm_Coarseness'] |
| recurrence | LargestAndVolumeTot | ['log-sigma-4-mm-3D_gldm_DependenceNonUniformityNormalized', 'log-sigma-2-mm-3D_firstorder_Maximum', 'wavelet-HHL_glcm_ClusterShade', 'original_glszm_LowGrayLevelZoneEmphasis', 'log-sigma-3-mm-3D_firstorder_Kurtosis', 'wavelet-LLH_glszm_SizeZoneNonUniformityNormalized', 'wavelet-LHH_firstorder_Median', 'log-sigma-3-mm-3D_ngtdm_Coarseness', 'wavelet-HLL_glszm_LargeAreaLowGrayLevelEmphasis', 'wavelet-LHL_firstorder_Skewness', 'wavelet-LHH_ngtdm_Contrast', 'wavelet-HHL_firstorder_Kurtosis', 'wavelet-HLH_glszm_SmallAreaLowGrayLevelEmphasis', 'wavelet-HHL_glcm_MCC', 'original_shape_Flatness', 'VolSum', 'log-sigma-2-mm-3D_glszm_ZonePercentage', 'log-sigma-3-mm-3D_glszm_LargeAreaLowGrayLevelEmphasis', 'wavelet-HLH_glszm_LargeAreaHighGrayLevelEmphasis', 'wavelet-LHH_ngtdm_Strength', 'wavelet-LHH_glszm_LargeAreaLowGrayLevelEmphasis', 'wavelet-HHL_glszm_LargeAreaHighGrayLevelEmphasis', 'log-sigma-5-mm-3D_glszm_SmallAreaEmphasis', 'wavelet-LHL_glszm_SmallAreaEmphasis', 'wavelet-LHH_glszm_ZoneVariance', 'log-sigma-3-mm-3D_glszm_SmallAreaEmphasis', 'wavelet-HLL_ngtdm_Strength', 'wavelet-LLH_glszm_LargeAreaLowGrayLevelEmphasis', 'log-sigma-5-mm-3D_ngtdm_Coarseness'] |
| recurrence | UnweightedAverage | ['log-sigma-4-mm-3D_gldm_DependenceNonUniformityNormalized', 'log-sigma-3-mm-3D_glszm_LargeAreaLowGrayLevelEmphasis', 'log-sigma-3-mm-3D_firstorder_Kurtosis', 'log-sigma-5-mm-3D_glszm_LargeAreaHighGrayLevelEmphasis', 'log-sigma-1-mm-3D_ngtdm_Contrast', 'wavelet-LLL_glszm_SizeZoneNonUniformityNormalized', 'original_shape_Elongation', 'wavelet-HHL_glcm_Imc1', 'wavelet-HHH_glszm_SmallAreaLowGrayLevelEmphasis', 'wavelet-HLL_glszm_LargeAreaHighGrayLevelEmphasis', 'log-sigma-2-mm-3D_glszm_SmallAreaLowGrayLevelEmphasis', 'log-sigma-5-mm-3D_ngtdm_Coarseness', 'wavelet-HHH_glcm_SumSquares', 'wavelet-HHH_gldm_DependenceNonUniformityNormalized', 'wavelet-HLH_glszm_ZoneVariance', 'log-sigma-5-mm-3D_ngtdm_Strength', 'log-sigma-4-mm-3D_glszm_SmallAreaEmphasis', 'wavelet-LHL_glszm_SmallAreaEmphasis', 'wavelet-LLH_ngtdm_Strength', 'log-sigma-3-mm-3D_ngtdm_Strength', 'log-sigma-2-mm-3D_firstorder_Maximum', 'wavelet-LHH_glszm_LargeAreaLowGrayLevelEmphasis'] |
| recurrence | WeightedAverage | ['log-sigma-3-mm-3D_glszm_ZonePercentage', 'wavelet-LLH_ngtdm_Strength', 'log-sigma-5-mm-3D_glszm_ZoneVariance', 'wavelet-HLL_ngtdm_Strength', 'log-sigma-2-mm-3D_glszm_SmallAreaLowGrayLevelEmphasis', 'original_shape_MinorAxisLength', 'wavelet-LHH_ngtdm_Contrast', 'wavelet-LHH_ngtdm_Strength', 'log-sigma-5-mm-3D_glszm_LargeAreaHighGrayLevelEmphasis', 'wavelet-LHL_firstorder_Kurtosis', 'log-sigma-5-mm-3D_ngtdm_Strength', 'wavelet-LHL_firstorder_Skewness', 'wavelet-HLH_glszm_LargeAreaHighGrayLevelEmphasis', 'wavelet-LHH_firstorder_Median', 'wavelet-HHL_glcm_ClusterShade', 'wavelet-LLH_glszm_LargeAreaLowGrayLevelEmphasis', 'wavelet-HLL_glszm_LargeAreaLowGrayLevelEmphasis', 'log-sigma-2-mm-3D_ngtdm_Contrast', 'wavelet-HLH_glszm_LargeAreaLowGrayLevelEmphasis', 'log-sigma-5-mm-3D_ngtdm_Coarseness', 'log-sigma-3-mm-3D_glszm_SmallAreaLowGrayLevelEmphasis', 'wavelet-LHH_glszm_LargeAreaLowGrayLevelEmphasis', 'log-sigma-3-mm-3D_ngtdm_Coarseness', 'wavelet-LHH_firstorder_Kurtosis', 'wavelet-LLL_gldm_LargeDependenceLowGrayLevelEmphasis'] |
| recurrence | WeightedAverage3L | ['log-sigma-2-mm-3D_glszm_ZonePercentage', 'wavelet-LLH_ngtdm_Strength', 'wavelet-LHL_ngtdm_Strength', 'wavelet-HLL_ngtdm_Strength', 'wavelet-LHH_glszm_ZoneVariance', 'log-sigma-3-mm-3D_glszm_LargeAreaLowGrayLevelEmphasis', 'original_shape_Maximum2DDiameterColumn', 'log-sigma-5-mm-3D_glszm_LargeAreaHighGrayLevelEmphasis', 'log-sigma-5-mm-3D_ngtdm_Strength', 'wavelet-HLH_glszm_LargeAreaHighGrayLevelEmphasis', 'log-sigma-3-mm-3D_ngtdm_Coarseness', 'wavelet-LHL_firstorder_Kurtosis', 'wavelet-HLL_glszm_LargeAreaLowGrayLevelEmphasis', 'wavelet-LHH_firstorder_Median', 'wavelet-LHL_firstorder_Skewness', 'wavelet-HLH_glszm_LargeAreaLowGrayLevelEmphasis', 'wavelet-HHL_glcm_ClusterShade', 'wavelet-LHH_glszm_LargeAreaLowGrayLevelEmphasis', 'log-sigma-5-mm-3D_ngtdm_Coarseness', 'wavelet-LHH_firstorder_Kurtosis', 'log-sigma-1-mm-3D_glcm_ClusterProminence', 'log-sigma-3-mm-3D_glszm_SmallAreaLowGrayLevelEmphasis', 'wavelet-LLL_gldm_LargeDependenceLowGrayLevelEmphasis', 'wavelet-LHH_ngtdm_Contrast'] |
| recurrence | Smallest | ['wavelet-LHH_gldm_DependenceNonUniformityNormalized', 'wavelet-HHH_glszm_SmallAreaLowGrayLevelEmphasis', 'wavelet-LLH_glcm_DifferenceVariance', 'wavelet-LLL_glszm_SizeZoneNonUniformityNormalized', 'wavelet-LLL_firstorder_Skewness', 'original_glcm_MCC', 'wavelet-LHH_glszm_SmallAreaLowGrayLevelEmphasis', 'wavelet-LHL_glszm_SmallAreaEmphasis', 'wavelet-HHH_firstorder_Skewness', 'wavelet-HHL_firstorder_Kurtosis', 'wavelet-HHL_glszm_SizeZoneNonUniformityNormalized', 'wavelet-HLL_glszm_SizeZoneNonUniformityNormalized', 'wavelet-LHL_glcm_InverseVariance', 'wavelet-HLH_glcm_InverseVariance'] |
| survival | Largest | ['log-sigma-4-mm-3D_gldm_DependenceNonUniformityNormalized', 'log-sigma-2-mm-3D_firstorder_Maximum', 'wavelet-HHL_glcm_ClusterShade', 'original_glszm_LowGrayLevelZoneEmphasis', 'log-sigma-3-mm-3D_firstorder_Kurtosis', 'wavelet-LLH_glszm_SizeZoneNonUniformityNormalized', 'wavelet-LHH_firstorder_Median', 'log-sigma-3-mm-3D_ngtdm_Coarseness', 'wavelet-HLL_glszm_LargeAreaLowGrayLevelEmphasis', 'wavelet-LHL_firstorder_Skewness', 'wavelet-LHH_ngtdm_Contrast', 'wavelet-HHL_firstorder_Kurtosis', 'wavelet-HLH_glszm_SmallAreaLowGrayLevelEmphasis', 'wavelet-HHL_glcm_MCC', 'original_shape_Flatness', 'log-sigma-2-mm-3D_glszm_ZonePercentage', 'log-sigma-3-mm-3D_glszm_LargeAreaLowGrayLevelEmphasis', 'wavelet-HLH_glszm_LargeAreaHighGrayLevelEmphasis', 'wavelet-LHH_glszm_LargeAreaLowGrayLevelEmphasis', 'wavelet-HHL_glszm_LargeAreaHighGrayLevelEmphasis', 'log-sigma-5-mm-3D_glszm_SmallAreaEmphasis', 'wavelet-LHL_glszm_SmallAreaEmphasis', 'log-sigma-3-mm-3D_glszm_SmallAreaEmphasis', 'wavelet-HLL_ngtdm_Strength', 'wavelet-LHH_glszm_ZoneVariance', 'wavelet-LHH_ngtdm_Strength', 'wavelet-LLH_glszm_LargeAreaLowGrayLevelEmphasis', 'log-sigma-5-mm-3D_ngtdm_Coarseness'] |
| survival | LargestAndNlesions | ['log-sigma-4-mm-3D_gldm_DependenceNonUniformityNormalized', 'log-sigma-2-mm-3D_firstorder_Maximum', 'wavelet-HHL_glcm_ClusterShade', 'original_glszm_LowGrayLevelZoneEmphasis', 'log-sigma-3-mm-3D_firstorder_Kurtosis', 'wavelet-LLH_glszm_SizeZoneNonUniformityNormalized', 'wavelet-LHH_firstorder_Median', 'log-sigma-2-mm-3D_glszm_ZonePercentage', 'wavelet-LHH_glszm_ZoneVariance', 'log-sigma-3-mm-3D_glszm_LargeAreaLowGrayLevelEmphasis', 'log-sigma-3-mm-3D_ngtdm_Coarseness', 'wavelet-HLH_glszm_LargeAreaHighGrayLevelEmphasis', 'wavelet-LHH_ngtdm_Strength', 'wavelet-HLL_glszm_LargeAreaLowGrayLevelEmphasis', 'wavelet-HLL_ngtdm_Strength', 'wavelet-LHL_firstorder_Skewness', 'wavelet-LHH_glszm_LargeAreaLowGrayLevelEmphasis', 'wavelet-HHL_glszm_LargeAreaHighGrayLevelEmphasis', 'wavelet-LHH_ngtdm_Contrast', 'log-sigma-3-mm-3D_glszm_SmallAreaEmphasis', 'wavelet-HHL_firstorder_Kurtosis', 'wavelet-HLH_glszm_SmallAreaLowGrayLevelEmphasis', 'wavelet-HHL_glcm_MCC', 'wavelet-LHL_glszm_SmallAreaEmphasis', 'original_shape_Flatness', 'Nlesions', 'log-sigma-5-mm-3D_glszm_SmallAreaEmphasis', 'wavelet-LLH_glszm_LargeAreaLowGrayLevelEmphasis', 'log-sigma-5-mm-3D_ngtdm_Coarseness'] |
| survival | LargestAndVolumeTot | ['log-sigma-4-mm-3D_gldm_DependenceNonUniformityNormalized', 'log-sigma-2-mm-3D_firstorder_Maximum', 'wavelet-HHL_glcm_ClusterShade', 'original_glszm_LowGrayLevelZoneEmphasis', 'log-sigma-3-mm-3D_firstorder_Kurtosis', 'wavelet-LLH_glszm_SizeZoneNonUniformityNormalized', 'wavelet-LHH_firstorder_Median', 'log-sigma-3-mm-3D_ngtdm_Coarseness', 'wavelet-HLL_glszm_LargeAreaLowGrayLevelEmphasis', 'wavelet-LHL_firstorder_Skewness', 'wavelet-LHH_ngtdm_Contrast', 'wavelet-HHL_firstorder_Kurtosis', 'wavelet-HLH_glszm_SmallAreaLowGrayLevelEmphasis', 'wavelet-HHL_glcm_MCC', 'original_shape_Flatness', 'VolSum', 'log-sigma-2-mm-3D_glszm_ZonePercentage', 'log-sigma-3-mm-3D_glszm_LargeAreaLowGrayLevelEmphasis', 'wavelet-HLH_glszm_LargeAreaHighGrayLevelEmphasis', 'wavelet-LHH_ngtdm_Strength', 'wavelet-LHH_glszm_LargeAreaLowGrayLevelEmphasis', 'wavelet-HHL_glszm_LargeAreaHighGrayLevelEmphasis', 'log-sigma-5-mm-3D_glszm_SmallAreaEmphasis', 'wavelet-LHL_glszm_SmallAreaEmphasis', 'wavelet-LHH_glszm_ZoneVariance', 'log-sigma-3-mm-3D_glszm_SmallAreaEmphasis', 'wavelet-HLL_ngtdm_Strength', 'wavelet-LLH_glszm_LargeAreaLowGrayLevelEmphasis', 'log-sigma-5-mm-3D_ngtdm_Coarseness'] |
| survival | UnweightedAverage | ['log-sigma-4-mm-3D_gldm_DependenceNonUniformityNormalized', 'log-sigma-3-mm-3D_glszm_LargeAreaLowGrayLevelEmphasis', 'log-sigma-3-mm-3D_firstorder_Kurtosis', 'log-sigma-5-mm-3D_glszm_LargeAreaHighGrayLevelEmphasis', 'log-sigma-1-mm-3D_ngtdm_Contrast', 'wavelet-LLL_glszm_SizeZoneNonUniformityNormalized', 'original_shape_Elongation', 'wavelet-HHL_glcm_Imc1', 'wavelet-HHH_glszm_SmallAreaLowGrayLevelEmphasis', 'wavelet-HLL_glszm_LargeAreaHighGrayLevelEmphasis', 'log-sigma-2-mm-3D_glszm_SmallAreaLowGrayLevelEmphasis', 'log-sigma-5-mm-3D_ngtdm_Coarseness', 'wavelet-HHH_glcm_SumSquares', 'wavelet-HHH_gldm_DependenceNonUniformityNormalized', 'wavelet-HLH_glszm_ZoneVariance', 'log-sigma-5-mm-3D_ngtdm_Strength', 'log-sigma-4-mm-3D_glszm_SmallAreaEmphasis', 'wavelet-LHL_glszm_SmallAreaEmphasis', 'wavelet-LLH_ngtdm_Strength', 'log-sigma-3-mm-3D_ngtdm_Strength', 'log-sigma-2-mm-3D_firstorder_Maximum', 'wavelet-LHH_glszm_LargeAreaLowGrayLevelEmphasis'] |
| survival | WeightedAverage | ['log-sigma-3-mm-3D_glszm_ZonePercentage', 'wavelet-LLH_ngtdm_Strength', 'log-sigma-5-mm-3D_glszm_ZoneVariance', 'wavelet-HLL_ngtdm_Strength', 'log-sigma-2-mm-3D_glszm_SmallAreaLowGrayLevelEmphasis', 'original_shape_MinorAxisLength', 'wavelet-LHH_ngtdm_Contrast', 'wavelet-LHH_ngtdm_Strength', 'log-sigma-5-mm-3D_glszm_LargeAreaHighGrayLevelEmphasis', 'wavelet-LHL_firstorder_Kurtosis', 'log-sigma-5-mm-3D_ngtdm_Strength', 'wavelet-LHL_firstorder_Skewness', 'wavelet-HLH_glszm_LargeAreaHighGrayLevelEmphasis', 'wavelet-LHH_firstorder_Median', 'wavelet-HHL_glcm_ClusterShade', 'wavelet-LLH_glszm_LargeAreaLowGrayLevelEmphasis', 'wavelet-HLL_glszm_LargeAreaLowGrayLevelEmphasis', 'log-sigma-2-mm-3D_ngtdm_Contrast', 'wavelet-HLH_glszm_LargeAreaLowGrayLevelEmphasis', 'log-sigma-5-mm-3D_ngtdm_Coarseness', 'log-sigma-3-mm-3D_glszm_SmallAreaLowGrayLevelEmphasis', 'wavelet-LHH_glszm_LargeAreaLowGrayLevelEmphasis', 'log-sigma-3-mm-3D_ngtdm_Coarseness', 'wavelet-LHH_firstorder_Kurtosis', 'wavelet-LLL_gldm_LargeDependenceLowGrayLevelEmphasis'] |
| survival | WeightedAverage3L | ['log-sigma-2-mm-3D_glszm_ZonePercentage', 'wavelet-LLH_ngtdm_Strength', 'wavelet-LHL_ngtdm_Strength', 'wavelet-HLL_ngtdm_Strength', 'wavelet-LHH_glszm_ZoneVariance', 'log-sigma-3-mm-3D_glszm_LargeAreaLowGrayLevelEmphasis', 'original_shape_Maximum2DDiameterColumn', 'log-sigma-5-mm-3D_glszm_LargeAreaHighGrayLevelEmphasis', 'log-sigma-5-mm-3D_ngtdm_Strength', 'wavelet-HLH_glszm_LargeAreaHighGrayLevelEmphasis', 'log-sigma-3-mm-3D_ngtdm_Coarseness', 'wavelet-LHL_firstorder_Kurtosis', 'wavelet-HLL_glszm_LargeAreaLowGrayLevelEmphasis', 'wavelet-LHH_firstorder_Median', 'wavelet-LHL_firstorder_Skewness', 'wavelet-HLH_glszm_LargeAreaLowGrayLevelEmphasis', 'wavelet-HHL_glcm_ClusterShade', 'wavelet-LHH_glszm_LargeAreaLowGrayLevelEmphasis', 'log-sigma-5-mm-3D_ngtdm_Coarseness', 'wavelet-LHH_firstorder_Kurtosis', 'log-sigma-1-mm-3D_glcm_ClusterProminence', 'log-sigma-3-mm-3D_glszm_SmallAreaLowGrayLevelEmphasis', 'wavelet-LLL_gldm_LargeDependenceLowGrayLevelEmphasis', 'wavelet-LHH_ngtdm_Contrast'] |
| survival | Smallest | ['wavelet-LHH_gldm_DependenceNonUniformityNormalized', 'wavelet-HHH_glszm_SmallAreaLowGrayLevelEmphasis', 'wavelet-LLH_glcm_DifferenceVariance', 'wavelet-LLL_glszm_SizeZoneNonUniformityNormalized', 'wavelet-LLL_firstorder_Skewness', 'original_glcm_MCC', 'wavelet-LHH_glszm_SmallAreaLowGrayLevelEmphasis', 'wavelet-LHL_glszm_SmallAreaEmphasis', 'wavelet-HHH_firstorder_Skewness', 'wavelet-HHL_firstorder_Kurtosis', 'wavelet-HHL_glszm_SizeZoneNonUniformityNormalized', 'wavelet-HLL_glszm_SizeZoneNonUniformityNormalized', 'wavelet-LHL_glcm_InverseVariance', 'wavelet-HLH_glcm_InverseVariance'] |
